# Philately as a Hobby for Medical Men

**Published:** 1912-06-22

**Authors:** C. J. Simpson


### RELAXATIONS AND HOBBIES.

### Philately as a Hobby for Medical Men.

By C. J. SIMPSON, B.A., M.B.

The majority of us get little or no opportunities to go into the country to study bird life and Nature in general, no matter how much we would like to do so—especially after reading articles such as those which appeared in The Hospital a few months ago. But it is when the long winter nights are on us that I can recommend philately. I know nothing more soothing and relaxing after a hard day's work than to get deep into one's stamps.

Philately, or the science of stamp collecting, is no new thing; in fact, there are very few of us who have not dabbled in it in our earlier days. In those days, however, we did not go in for philately; we simply went in for accumulating stamps—quantity was our motto. Now we are older this does not satisfy us, and so we go in for the science of stamp-collecting, *i.e.*, quality as well as quantity.

### The Stamp and the Schoolboy.

Apart from the mere intrinsic value of stamps they have a decided educational value. Look how it helped us with our geography at school—how often we consulted our Map of the World to find out where some particular place was, so that we could put a stamp from there in its proper place in the album; thus we found Caicos Island to be part of the Bahamas, the Seychelles off the east coast of Africa, etc. Van Diemen's Land we searched for in vain until we found it was the old name for Tasmania. So gradually we got to know where various out-of-the-way spots were, and, what is more, we knew to what Power they belonged, for most stamp albums are arranged giving the great Powers and their various colonies and dependencies in each continent.

Another thing is the coinage of each country. We soon get to know the different coinages, and more useful still, their relative values, as according to agreement most countries now have stamps of equivalent values printed in the same colour: thus halfpenny denominations are in green, penny in red, twopence-halfpenny in blue, and thus we see at once that 5 cents United States, 10 centavos Mexico, 25 centimes France, 25 pfennig Germany, 1 piastre Egypt, and $2\frac{1}{2}$ pence England are all equivalent, and from this it follows that the Mexican dollar (peso) is only worth half a United States dollar.

The history of each country is reflected on its stamps. Take, let us say, the Transvaal, formerly the South African Republic. Until 1877 we get the stamps of the first Republic with the arms in the centre and all names and values in Dutch; then we get these stamps during the first British occupation in July of that year overprinted with V.R. Transvaal; then next year, 1878, we get an issue with a portrait of Queen Victoria, values, etc., in English. Then in 1882 we get the second Republic, and this 1878 issue is now surcharged with the values in Dutch; then we get a reissue of the first Republic stamps, followed soon after by a new issue of the second Republic, which lasts with minor alterations until 1900. During the second British occupation the Dutch stamps are surcharged V.R.I., and later on, after the death of the Queen, E.R.I., and later still in 1902, we get the general issue with King Edward VII.'s portrait. Look at the history given in this brief sketch—think of other countries and how events there leave similar imprints on their stamps; as soon as a new king or president is elected out comes his portrait on the stamps of that country. The various striking events in the past history of a country are often well shown in some of the commemorative issues, *e.g.*, the Columbus issue of the United States. The various resources of the country may also be shown, as in the Newfoundland issue, where you get pictures of mining, fishing, timber, etc., all shown to you as forming part of its resources.

### A Question of Sentiment.

Enough, I think, to show the educational value of stamps; now to turn to another aspect—that of keeping up our friendships. We all know how soon after we were qualified the various men of our year and college chums drifted out of sight, one joined the Royal Army Medical Corps, another the Indian Medical Service, and so they go. We are busy with our own affairs and never remember to drop them a few lines to see how they are getting on—perhaps we even forget their existence. But if we still kept up our stamps we would always have a reminder of other places—say we are putting in some Hong Kong stamps into our album. At once we think if Smith, R.N., is still out there and how he is getting on, and so next mail a letter goes out to Hong Kong with a request, of course, for some stamps from Smith's local letters. Back comes a letter saying how good it is to hear from an old pal when cruising up some God-forsaken river a hundred miles from nowhere, and with only yourself and the few officers of the gunboat for company, and so our friendships are kept up.

As I said before, stamps have a commercial aspect, and so we may regard them not only as a hobby but also more or less as an investment, and if properly managed a very safe investment too, with the great advantage that, like wine, they improve with age, so that when we get tired of our collection and put it aside, when we come across it again a few years later it is not only none the worse, but increased in value.

This is the age of specialisation both for stamps as well as for medicine. Owing to the immense number of new issues constantly coming out we cannot keep up with them all in a general collection, and so insensibly we drift into specialising in some countries we have a fancy for, or may happen to get more stamps of.

### How to Begin in Later Life.

To begin with, it is best to start with a general collection, and then when we find it getting uncomfortably big to go in for groups of countries, continents, etc., as we have the fancy, *e.g.*, Great Britain and Colonies, or stamps of King Edward VII., or stamps of Asia, etc. The first thing to do is to get a good nucleus to start on. If you have the old collection of your schooldays, well and good. You may find some fairly good stamps in it, though probably not the ones you thought most of when at school! If you have not an old one to start on the best thing is to buy a collection second-hand—these are constantly offered for sale in the *Bazaar, Exchange and Mart*—and a collection of, say, 1,600 to 1,800 stamps stuck in with mounts in an ordinary album in good condition should not cost more than twenty shillings.

Having got your nucleus you wish to add to it, and here you make use of your various friends abroad. These you write to and ask for any loose stamps they may come across, especially the higher values which arrive on parcels, etc.; also any business friend with a large foreign correspondence is a help. From all these sources you will soon have a lot of stamps which you sort into their countries, and those varieties not already in your album you stick in, using thin hinges of gummed paper in doing so (these stamp-mounts you can buy from any stamp dealer or shop at 6d. per 1,000 or less); the others are now your duplicates which you wish to exchange for other varieties which you have not got. At this stage it would be advisable to get a stamp catalogue so that you may have a rough idea not only of the catalogue value of stamps but also the dates of their respective issues and the number of stamps in each issue, so that when arranging each issue in your album you can leave gaps for the various values you have still to get. The catalogues most used are Stanley Gibbons' Parts I. and II., 2s. 6d. each net, and Bright's ABC, costing for adhesive postage stamps alone, I think, 2s.

The easiest way to exchange is to do so with any friends you may have who collect, and here your knowledge of the catalogued value of stamps will keep you pretty right. Later on when you have exhausted your friends' exchange resources, you want to find another outlet for your duplicates, and the best thing perhaps to do now is to join a stamp club. These are always being advertised, and their plan is, with slight modifications, the same—as a rule, a nominal subscription of a few shillings to cover the cost of working expenses. Then each member gets one or more sheets sent to him in which he sticks his duplicates and prices them according to the catalogue the club uses. These sheets are then sent round the various members in turn, and each takes off just whatever stamps he needs and passes it on to the next on the list, and so on. At the end of each month the sheets are balanced, and each member pays the difference between the stamps he has taken off the various sheets and those taken off his. Usually only half this difference is paid, *i.e.*, he gets his stamps at half catalogue prices. So if your duplicates are any good it may cost you little more than the postage. If you think you cannot get enough stamps to choose from this way, any dealer will only be too glad to send you stamps on approval, but I think you will find the exchange plan to be better—it certainly is cheaper. Another very good way is to exchange with collectors abroad who sometimes advertise in the papers here, but doing so is risky unless they give references or you know them. Here you send out stamps of your country and they send back equal values of theirs. Properly done this is the best way to get rid of duplicates.

### What Stamps to Avoid.

As to what *not* to collect. A good rule is never to put in your album a torn stamp or one that has had its perforated edge cut off, for besides taking away from the look of your album its value is nil. If you happen to get one given to you, or in your duplicates you might put it in provisionally till you can get a better copy, but it is better never to put them in at all—certainly never buy or exchange one, no matter how cheap it may appear to be for an otherwise valuable stamp, for as a rule it is always the valuable stamps that seem to get torn—worse luck! Do not put in newspaper wrapper or postcard stamps or fiscals; in fact, confine yourself to adhesive stamps used for postal purposes, which you will find will give you plenty of scope. As for reprints, always fight shy of unused stamps, especially those of some of the Central and South American States—personally I make it a rule only to get postally used copies unless I happen to know where the stamps really came from.

Regarding varieties of perforation and watermark, these you will only get into in the course of time, but your catalogue will help you here. When you get to know them and are able and desirous of differentiating them, then it is time for you to specialise, for this multiplies the varieties of the stamps of each country tremendously so as to be quite beyond the scope of the average general collector.

